# The complete mitochondrial genome of *Crematogaster matsumurai* (Forel 1901) (Hymenoptera: Formicidae) and its phylogenetic relationship

**DOI:** 10.1080/23802359.2022.2100291

**Published:** 2022-07-28

**Authors:** Jin-chao Wang, Jiang-hui Cheng, Li-min Chen, Xiao-wei Li, Hai-xia Zhang, Qiang-gen Zhu, Ai-wu Jin

**Affiliations:** a College of forestry and Biotechnology, Zhejiang Agriculture and Forestry University, Hangzhou, China; b Integrated Plant Protection Center, Lishui Academy of Agricultural and Forestry Sciences, Lishui, China; c College of Ecology, Lishui University, Lishui, China; d State Key Laboratory for Managing Biotic and Chemical Threats to the Quality and Safety of Agro-products, Institute of Plant Protection and Microbiology, Zhejiang Academy of Agricultural Sciences, Hangzhou, China; e College of Chemistry and Life Sciences, Zhejiang Normal University, Jinhua, China

**Keywords:** *Crematogaster matsumurai*, complete mitochondrial genome, phylogeny, Hymenoptera, *Phyllostachys heterocycla* (Carr.)

## Abstract

*Crematogaster matsumurai* (Forel 1901) is an important arboreal ant species commonly found on *Phyllostachys heterocycla* (Carr.) in Lishui, Zhejiang, China. This study analyzed the mitochondrial genome sequence of *C. matsumurai* and discussed its phylogenetic relationship in Hymenoptera. The circular mitochondrial genome was 16,028 bp long, including a standard set of 22 transfer RNAs (tRNAs), two ribosomal RNAs (rRNAs), and 13 protein-coding genes (PCGs), which showed the typical insect mitochondrial genome arrangement. The AT and GC contents of the mitochondrial genome sequence were 76.92% and 23.08%, respectively. The maximum-likelihood (ML) phylogenetic analysis based on whole mitochondrial genome sequences showed that *C. matsumurai* is closest to *Crematogaster teranishii*.

*Crematogaster* (Hymenoptera: Formicidae: Myrmicinae) ants are diverse, widespread, and abundant in tropical, subtropical, and warm-temperate climates throughout the world (Blaimer 2012). Arboreal species of *Crematogaster* in particular are often dominant in the ant fauna, with polydomous and strongly territorial colonies (Dejean et al. 2010; Blaimer 2019). Species of this genus have a flat petiole and its postpetiole connected to the dorsal surface of the gaster, which is unique among all other Myrmicinae ants (Blaimer and Fisher 2013). *Crematogaster matsumurai* (Forel 1901) is an ant species commonly found in East Asia. It has been reported as a predator of some pests, such as *Halyomorpha halys* (Kamiyama et al. 2021). In the natural environment, this species mainly lives in decayed parts of relatively tall trees, such as *Acer palmatum*, *Prunus jamasakura*, and *Prunus yedoensis* (Harada 2005; Hosoishi et al. 2019), and is also commonly found on *Phyllostachys heterocycla* (Carr.) in Lishui, Zhejiang, China. It builds insect nets nests on the leaves, stems, and branches of *P. heterocycle* (Carr.) to catch insects, such as aphids. Moreover, *C. matsumurai* develops mutualistic associations with other organisms. Despite its local abundance and important ecological interactions, the phylogenetic relationship of *C. matsumurai* within genus *Crematogaster* is controversial and in great need of modern revisionary studies (Sharaf et al. 2019). Therefore, this paper studied the mitochondrial genome of *C. matsumurai* and analyzed the evolutionary relationship between species.

Adults of *C. matsumurai* were collected from *P. heterocycle* (Carr.) forest in Longquan City (N28°14′35.12″, E119°14′30.31″), Lishui City, Zhejiang Province, China, and preserved in pure ethanol. The collected samples were identified and stored at −40 °C in the Institute of Integrated Plant Protection Center, Lishui Academy of Agricultural and Forestry Sciences, Lishui, China (http://nky.lishui.gov.cn/, Jin-chao Wang, jcw199907@163.com) under the voucher number 20210805LQCM. Total genomic DNA was extracted by using E.Z.N.A.^®^ Insect DNA kit and applied to 300 bp paired-end library construction using the Truseq SBS Kit (300 cycles) for Illumina sequencing. Sequencing was carried out on the Illumina NovaSeq 6000 platform (Biozeron Co., Ltd., Shanghai, China). A total of 5449.1 Mb of raw reads were generated, and by employing the tool Trimmomatic v0.39, the reads having adapter contamination and the small pieces less than 75 bp in length after quality trimming were removed to obtain clean reads. De novo genome assembly were conducted by SPAdes v3.14.1. The mitochondrion genes were annotated using the online MITOS tool (Bernt et al. 2013), using default parameters to predict protein-coding genes (PCGs), transfer RNA (tRNA) genes, and ribosomal RNA (rRNA) genes. The genomic sequence has been deposited in GenBank with an accession number OM328370.

The complete mitochondrial genome of *C. matsumurai* was a typical circular DNA molecule of 16,028 bp in length. A total of 37 genes were annotated, including 13 PCGs, 22 tRNAs, and two rRNAs. The AT content of the whole genome was 76.92%. 12 PCGs began with ATN as the start codon, and the start codon of NAD1 was TTG. The COI, COII, COIII, ATP6, ATP8, NAD1, NAD2, NAD3, NAD4, NAD4L, NAD5, NAD6, and COB genes were terminated with TAA as the stop codon.

To reveal the phylogenetic relationship of *C. matsumurai* with other members in Hymenoptera, phylogenetic analysis was performed based on 13 mitochondrial PCGs, of which five species, *Frankliniella intonsa*, *Apolygus lucorum*, *Empoasca flavescens*, *Spodoptera frugiperda*, and *Tuta absoluta* were served as outgroups. Functional annotations were performed using sequence-similarity Blast searches with a typical cutoff *E*-value of 10^−5^ against several publicly available protein databases: NCBI non-redundant (Nr) protein database, Swiss-Prot, Clusters of Orthologous Groups (COGs), and Kyoto Encyclopedia of Genes and Genomes (KEGG) and Gene Ontology (GO) terms (Yin et al. 2012). The maximum-likelihood (ML) bootstrap analysis with 1000 replicates was performed using RaxML v8.2.12 (Stamatakis 2014). The phylogenetic tree showed that *C. matsumurai* was closely related to *C. teranishii* (Figure 1). Meanwhile, genus *Crematogaster* had a close relationship with *Ochetellus* in Hymenoptera. The genome sequence of *C. matsumurai* in this study might provide useful information for Hymenoptera researches.

**Figure 1.**
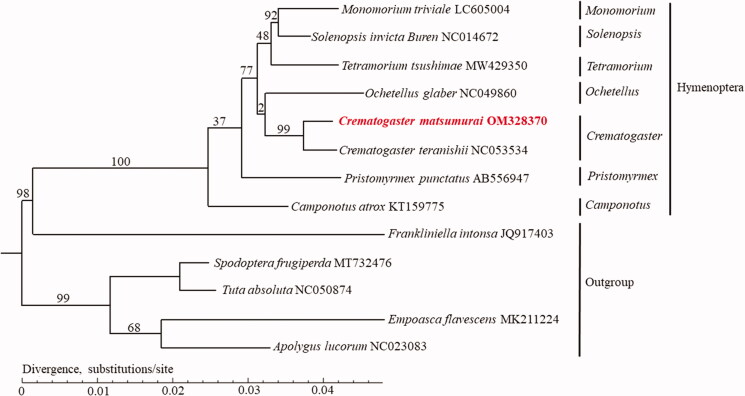
Phylogenetic tree of 13 insect species, including *Crematogaster matsumurai* based on the nucleotide dataset of the 13 mitochondrial protein-coding genes. The maximum-likelihood bootstrap values are indicated above nodes. The GenBank numbers and tribe of all species are shown in the figure.

## Data Availability

The genome sequence data that support the findings of this study are openly available in GenBank of NCBI at https://www.ncbi.nlm.nih.gov/ under the accession no. OM328370. The associated BioProject, SRA, and Bio-Sample numbers are PRJNA790703, SRP352081, and SAMN24220740, respectively.
